# Supplementary material for: Aging alters neural activity at event boundaries in the hippocampus and Posterior Medial network
Source: Nat Commun. 2020 Aug 7;11:3980. doi: 10.1038/s41467-020-17713-4 (PMC7414222; doi:10.1038/s41467-020-17713-4)
Supplement: Supplementary file 1 — Supplementary Information [file 41467_2020_17713_MOESM1_ESM.pdf]

Supplementary Information for: Aging alters neural activity at event boundaries in the hippocampus and Posterior Medial network

Short Title: Aging and neural event segmentation

Zachariah M. Reagh[1,2], Angelique Delarazan, Alexander Garber, & Charan Ranganath[2,3]

Author Affiliations:

[1] Department of Neurology, University of California, Davis

[2] UC Davis Center for Neuroscience, University of California, Davis

[3] Department of Psychology, University of California, Davis

Please address correspondence to:

Zachariah M. Reagh

UC Davis Center for Neuroscience

1544 Newton Court

Davis, CA 95618

zreagh@ucdavis.edu

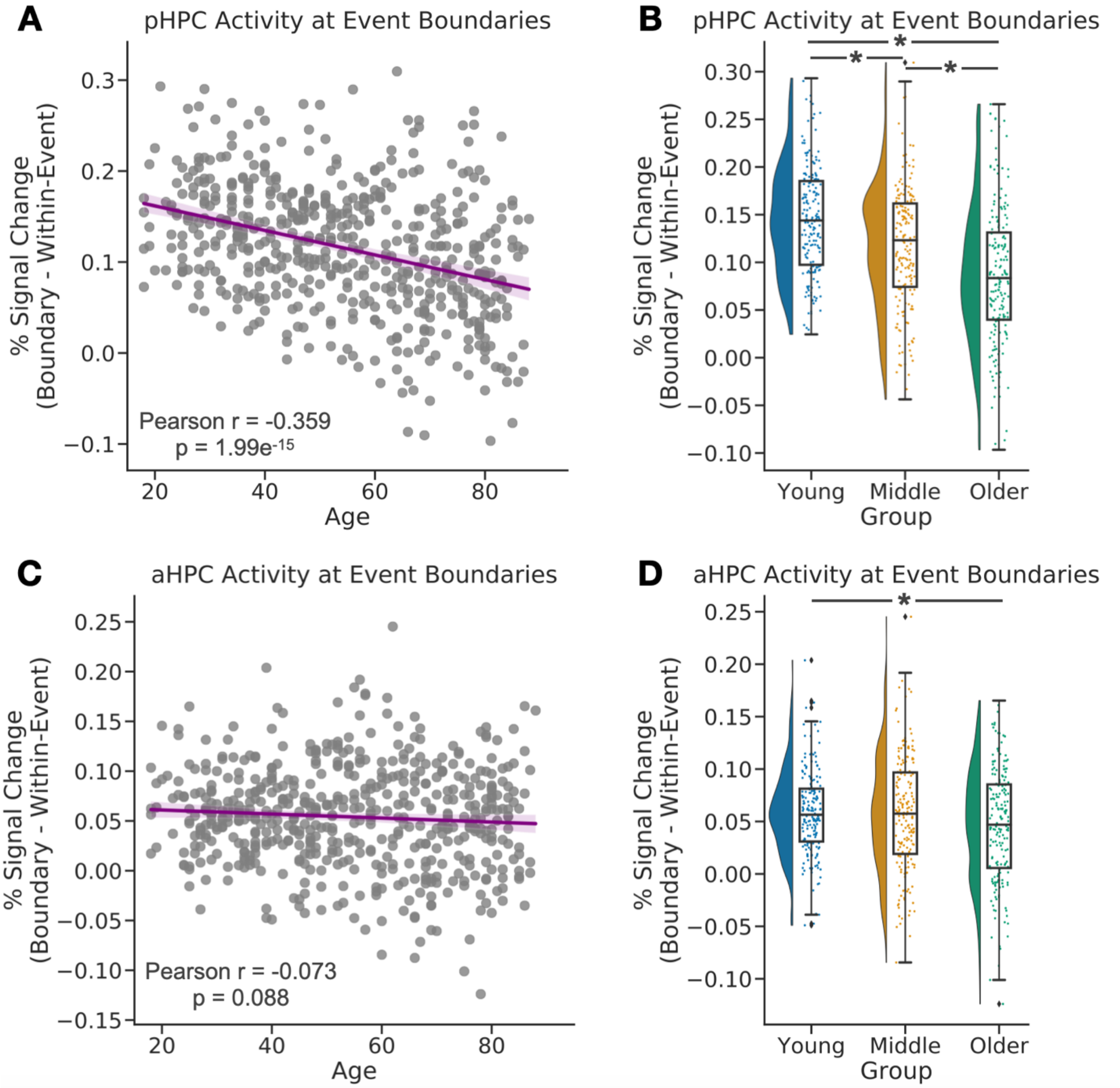

Supplementary Figure 1: Boundary-evoked activity and age-related decline in anterior and posterior hippocampus. Data are displayed as activity evoked by event boundaries, not adjusted for within-event activity as in the main text. (A) Activity at event boundaries significantly declines with age in pHPC. (B) This effect holds when grouping, and comparing across Young, Middle, and Older individuals. (C) Though a similar relationship is observed in aHPC, it does not reach statistical significance. (D) The Older adult group nonetheless shows significantly lower activity than the Young group. (Correlations were assessed via Person r. * indicates a significant difference via Tukey's HSD at $p < 0.05$ corrected following a one-way ANOVA. Raincloud plots depict data distributions across groups, with box components displaying median values and data quartiles. N=546 for correlations, N=182 per group for groupwise comparisons, examined over a single experiment.)

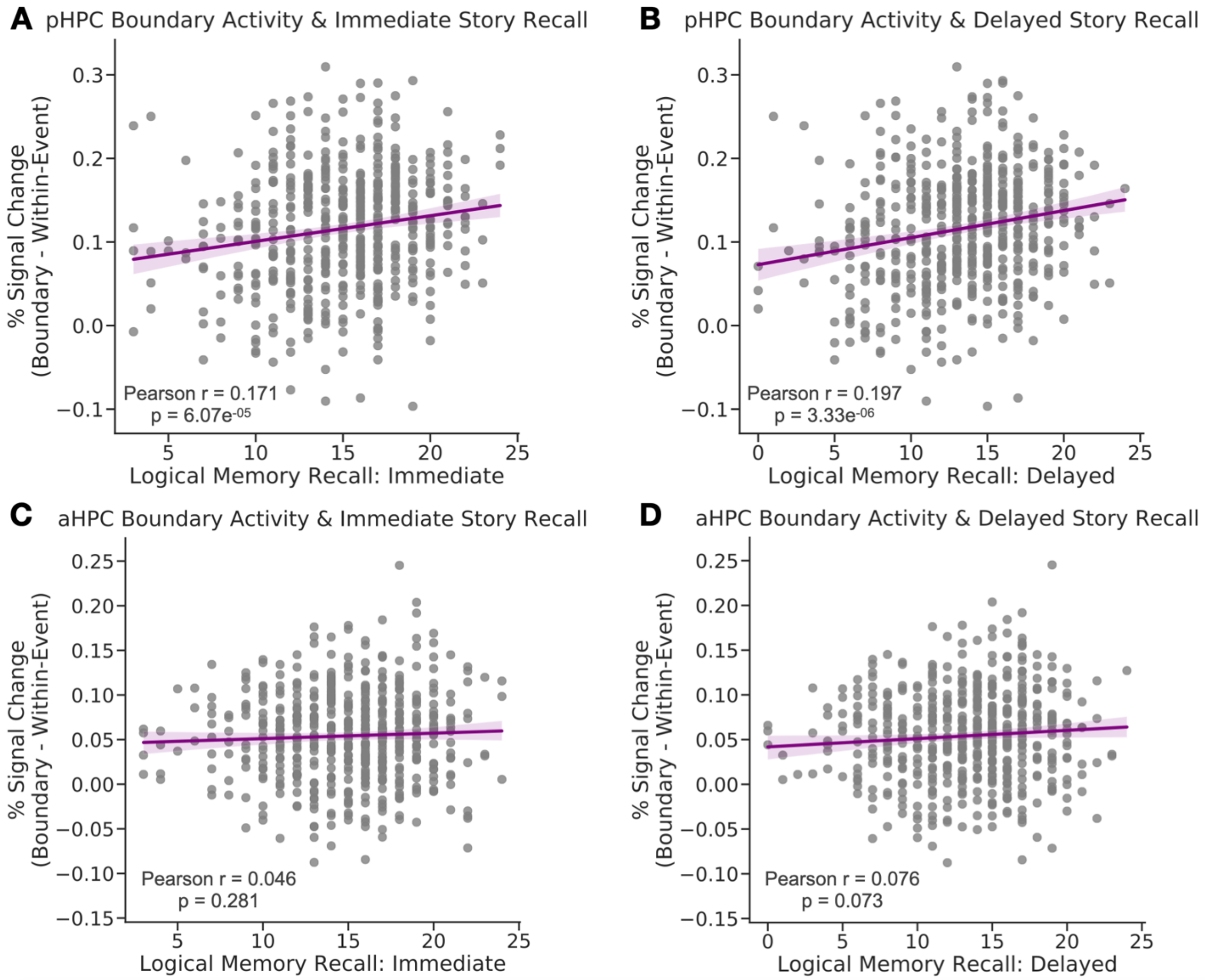

Supplementary Figure 2: Boundary-evoked activity and age-related decline in anterior and posterior hippocampus. Data are displayed as activity evoked by event boundaries, not adjusted for within-event activity as in the main text. pHPC activity at event boundaries significantly predicts memory for stories in immediate (A) and delayed (B) recall conditions. These relationships did not reach significance in aHPC (C,D). (Correlations were assessed via Person r.)

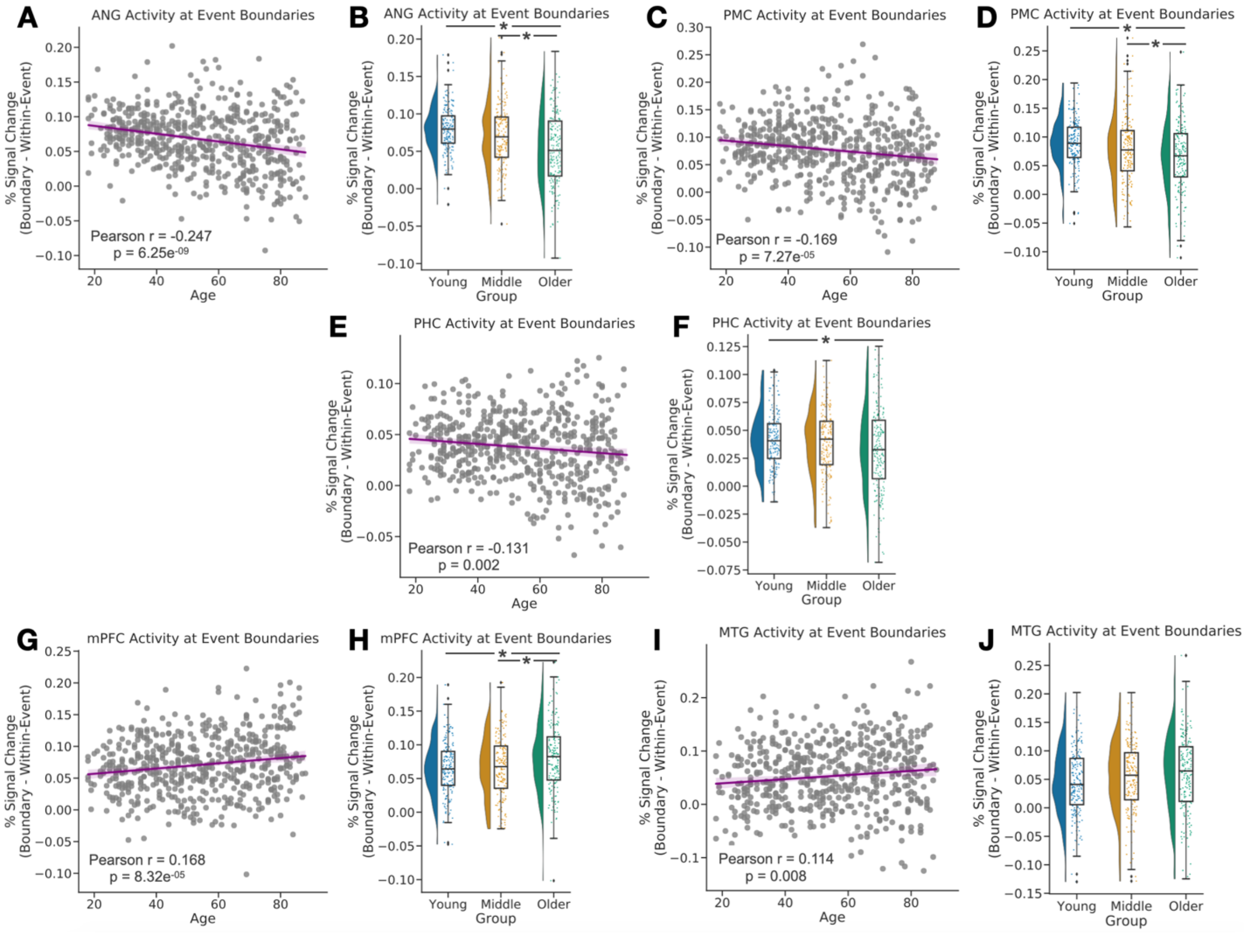

Supplementary Figure 3: Declines and increases in boundary-evoked activity in the posterior-medial network. Data are displayed as activity evoked by event boundaries, not adjusted for within-event activity as in the main text. Age-related declines in boundary-evoked responses in angular gyrus (A,B), posterior-medial cortex (C,D), and parahippocampal cortex (E,F). Age-related increases in boundary-evoked responses in medial prefrontal cortex (G,H) and middle temporal gyrus (I,J). (Correlations were assessed via Person r. * indicates a significant difference via Tukey's HSD at $p < 0.05$ corrected following a one-way ANOVA. Raincloud plots depict data distributions across groups, with box components displaying median values and data quartiles. N=546 for correlations, N=182 per group for groupwise comparisons, examined over a single experiment.)

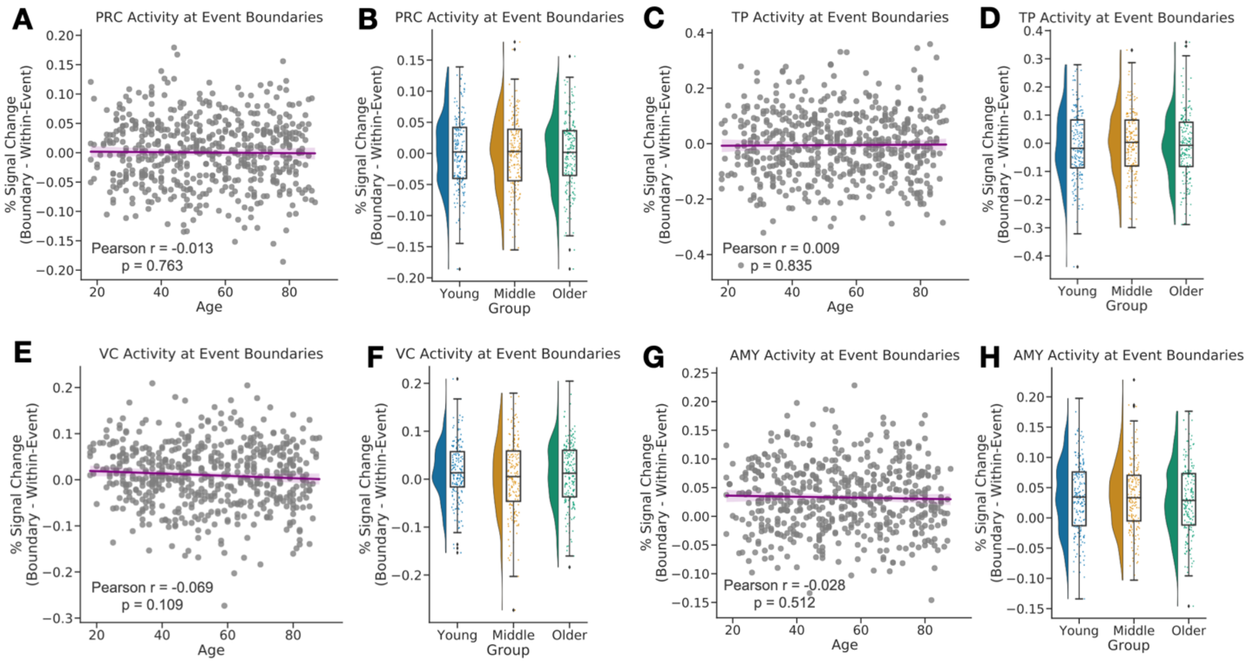

Supplementary Figure 4: Anterior-temporal regions and visual cortex do not show age-related changes. Data are displayed as activity evoked by event boundaries, not adjusted for within-event activity as in the main text. Boundary-evoked responses were not observed in (A,B) perirhinal cortex or (C,D) temporal poles. Boundary-evoked responses were observed in (E,F) visual cortex and (G,H) amygdala, but these did not significantly change with age. (Correlations were assessed via Person r. Raincloud plots depict data distributions across groups, with box components displaying median values and data quartiles. N=546 for correlations, N=182 per group for groupwise comparisons, examined over a single experiment.)
