# Supplementary material for: Aging alters neural activity at event boundaries in the hippocampus and Posterior Medial network
Source: Nat Commun. 2020 Aug 7;11:3980. doi: 10.1038/s41467-020-17713-4 (PMC7414222; doi:10.1038/s41467-020-17713-4)
Supplement: Supplementary file 3 — Reporting Summary [file 41467_2020_17713_MOESM3_ESM.pdf]

nature research

Corresponding author(s): Zachariah Reagh

Last updated by author(s): Mar 26, 2020

Reporting Summary

Nature Research wishes to improve the reproducibility of the work that we publish. This form provides structure for consistency and transparency in reporting. For further information on Nature Research policies, see Authors & Referees and the Editorial Policy Checklist.

Statistics

For all statistical analyses, confirm that the following items are present in the figure legend, table legend, main text, or Methods section.

n/a	Confirmed	
☐	☒	The exact sample size (n) for each experimental group/condition, given as a discrete number and unit of measurement
☐	☒	A statement on whether measurements were taken from distinct samples or whether the same sample was measured repeatedly
☐	☒	The statistical test(s) used AND whether they are one- or two-sided<br>*Only common tests should be described solely by name; describe more complex techniques in the Methods section.*
☐	☒	A description of all covariates tested
☐	☒	A description of any assumptions or corrections, such as tests of normality and adjustment for multiple comparisons
☐	☒	A full description of the statistical parameters including central tendency (e.g. means) or other basic estimates (e.g. regression coefficient) AND variation (e.g. standard deviation) or associated estimates of uncertainty (e.g. confidence intervals)
☐	☒	For null hypothesis testing, the test statistic (e.g. F, t, r) with confidence intervals, effect sizes, degrees of freedom and P value noted<br>*Give P values as exact values whenever suitable.*
☒	☐	For Bayesian analysis, information on the choice of priors and Markov chain Monte Carlo settings
☒	☐	For hierarchical and complex designs, identification of the appropriate level for tests and full reporting of outcomes
☐	☒	Estimates of effect sizes (e.g. Cohen's d, Pearson's r), indicating how they were calculated

Our web collection on statistics for biologists contains articles on many of the points above.

Software and code

Policy information about availability of computer code

Data collection	Boundary ratings were collected using PsychoPy version 1.85.0 by Ben-Yakov & Henson, 2018. We used the same software for our in-house collection of event boundary ratings. Software involved in presentation of the movie in the MRI scanner is not explicitly described in the publications relating to the release of the dataset (Shafto et al., 2014 or Taylor et al., 2017). Therefore, we are unfortunately unable to report this detail.
Data analysis	Bash v3.2.57, Python v3.7, Analysis of Functional Neuroimages (AFNI) v20.1.13, FreeSurfer v6.0, and Advanced Normalization Techniques (ANTs) v2.1.1 were used to analyze the data.

For manuscripts utilizing custom algorithms or software that are central to the research but not yet described in published literature, software must be made available to editors/reviewers. We strongly encourage code deposition in a community repository (e.g. GitHub). See the Nature Research guidelines for submitting code & software for further information.

Data

Policy information about availability of data

All manuscripts must include a data availability statement. This statement should provide the following information, where applicable:

- Accession codes, unique identifiers, or web links for publicly available datasets
- A list of figures that have associated raw data
- A description of any restrictions on data availability

Analytical code and fully processed quantitative data are available upon request to Z.M.R. To apply for access to raw behavioral and MRI data, a request must be made to the CamCAN group at https://camcan-archive.mrc-cbu.cam.ac.uk/dataaccess/.

Field-specific reporting

Please select the one below that is the best fit for your research. If you are not sure, read the appropriate sections before making your selection.

- [ ] Life sciences
- [x] Behavioural & social sciences
- [ ] Ecological, evolutionary & environmental sciences

For a reference copy of the document with all sections, see nature.com/documents/nr-reporting-summary-flat.pdf

Behavioural & social sciences study design

All studies must disclose on these points even when the disclosure is negative.

Study description

The study is a large sample fMRI data analysis using quantitative methods.

Research sample

Participants were drawn from the CamCAN dataset (http://www.camcan. org/index.php?content=dataset). The sample consisted of 623 participants (316 female) for whom all fMRI and neuropsychological data of interest was present. From this initial sample, 546 participants (271 female, mean age = 54.08, SD = 18.56, range = 18-88) were included in our analyses due to issues with neuroimaging data quality.

Sampling strategy

As described in Shafto et al., 2014, participants were recruited on a volunteer basis.

Data collection

As is described in Taylor et al., NeuroImage 2017:

All MRI datasets were collected at a single site (MRC-CBSU) using a 3 T Siemens TIM Trio scanner with a 32-channel head coil. Participants were scanned in a single 1-hour session. Before scanning, physiological measurements were taken, and two behavioural experiments were run. In the scanner, memory foam cushions were used for comfort and to minimise head movement. Instructions and visual stimuli for functional tasks were back-projected onto a screen viewed through a mirror mounted on the head coil; auditory stimuli were presented via MR-compatible etymotics headphones; and manual responses were made with the right hand using a custom-built MR-compatible button-box. Cardiac data were recorded using photoplethysmograph/pulse-oximeter on the left index finger, sampled at 50 Hz.

Members of the Cambridge Centre for Aging and Neuroscience and/or Cambridge Brain Sciences Unit were present during data collection. No indication of blinding is evident from Shafto et al., 2014, or Taylor et al., 2017, though this study features no between-subjects manipulations.

Timing

Data were collected between 2014-2017.

Data exclusions

The sample consisted of 623 participants (316 female) for whom all fMRI and neuropsychological data of interest was present. From this initial sample, 546 participants (271 female) were included in our analyses due to issues with neuroimaging data quality. These exclusions primarily pertained to movement in the MRI scanner, and a smaller number of participants had significant signal dropout in key brain regions.

Non-participation

No indication of participant dropout or non-participation is evident from Shafto et al., 2014 or Taylor et al., 2017.

Randomization

Participants in the experiment are drawn from the general population of the United Kingdom via medical practitioner registration. Thus, although this is a sample of convenience on a volunteer basis, participants are fairly representative of the broader UK demographic.

Reporting for specific materials, systems and methods

We require information from authors about some types of materials, experimental systems and methods used in many studies. Here, indicate whether each material, system or method listed is relevant to your study. If you are not sure if a list item applies to your research, read the appropriate section before selecting a response.

Materials & experimental systems

n/a	Involved in the study
[x]	[ ] Antibodies
[x]	[ ] Eukaryotic cell lines
[x]	[ ] Palaeontology
[x]	[ ] Animals and other organisms
[ ]	[x] Human research participants
[x]	[ ] Clinical data

Methods

n/a	Involved in the study
[x]	[ ] ChIP-seq
[x]	[ ] Flow cytometry
[ ]	[x] MRI-based neuroimaging

Human research participants

Policy information about studies involving human research participants

Population characteristics	The sample consisted of 623 participants (316 female) for whom all fMRI and neuropsychological data of interest was present. Participants ranged from 18-88 years in age, and demographics of the United Kingdom as of 2014 (see Shafto et al., 2014) were used to guide recruitment. Selection bias may be present in that distinguishing characteristics may exist in individuals more likely to volunteer for research studies. However, with such broad recruitment techniques and such a relatively large sample size, it is unlikely that this would significantly impact the results of our analyses.
Recruitment	Participants were recruited on a volunteer basis in partnership between Cambridge University and regional primary care providers.
Ethics oversight	Written informed consent was obtained in accordance with the Cambridgeshire Research Ethics Committee.

Note that full information on the approval of the study protocol must also be provided in the manuscript.

Magnetic resonance imaging

Experimental design

Design type	Event-related.
Design specifications	Data consisted of a single fMRI run with 24 trials of interest (12 per condition). Each trial was modeled with a finite impulse response (FIR) function with 6 'steps' modeled over an interval of 12 seconds. Trials were defined as event boundaries in a naturalistic stimulus, so timing was not controlled. However, the average time between events was approximately 29.2 seconds.
Behavioral performance measures	We analyzed select neuropsychological tests from the full battery included in the CamCAN protocol. Of particular interest to us were the Logical Memory immediate and delayed recall tests from the Wechsler Memory Scale, and the composite memory, fluency, and visuospatial performance scores from the Addenbrooke's Cognitive Examination (ACE M; ACE-F; ACE-VS).

Acquisition

Imaging type(s)	Functional
Field strength	3T
Sequence & imaging parameters	Functional data during movie viewing consisted of a T2*-weighted Echo Planar Imaging (EPI) sequence with the following parameters: TR = 2470ms; TE (5 echoes) = 9.4ms, 21.2ms, 33ms, 45ms, 57ms; flip angle = 78 degrees; 32 axial slices; slice thickness = 3.7mm with an interslice gap of 0.74mm (20% of slice thickness); FOV = 192x192mm; voxel size = 3x3x4.44mm; acquisition time = 8min, 13 sec.
Area of acquisition	Whole-brain.

Diffusion MRI ☐ Used ☒ Not used

Preprocessing

Preprocessing software	Data were preprocessed using AFNI (version 18.2.15; https://afni.nimh.nih.gov), ANTs (http://stnava.github.io/ANTs). AFNI preprocessing used the standardized afni_proc.py pipeline, with specific steps as follows: 1) despiking of the functional time series (3dDespike); 2) slice timing correction (3dTshift); 3) coregistration of functional to anatomical images (align_epi_anat.py); 4) motion correction with alignment to the minimum outlier in the time series (3dvolreg); 5) masking of the functional time series to brain voxels as defined by the anatomical image (3dcalc); 6) generation of tissue maps (gray matter, white matter, CSF); 7) normalization and scaling of each voxel time series and conversion to percent signal change (3dTstat, 3dcalc).
Normalization	Within-participant anatopical-EPI registration occurred via AFNI's align_epi_anat.py step, and all images were registered to MNI space. Subsequently, we constructed a group template from all 546 participants (antsMultivariateTemplateConstruction2.sh), and all analyses took place at the level of the group template (in MNI space).
Normalization template	MNI305 (see above).
Noise and artifact removal	We computed a high-frequency visual information vector was entered into our GLM as a regressor of non-interest (see 'Neuroimaging data analysis' in the manuscript for details). Twelve continuous nuisance regressors were also included to account for motion (6 motion regressors – x, y, z, pitch, roll, yaw – plus the derivative of each). These thirteen nuisance regressors served as the model baseline. We also included a nuisance regressor to account for linear drift in the fMRI signal.

Volume censoring	Participants whose average framewise displacement exceeded 1mm, or whose maximal framewise displacement exceeded 3mm (derived during motion correction) were excluded from analyses.

Statistical modeling & inference

Model type and settings	Our mass univariate fMRI model involved thirteen nuisance regressors (see Noise and artifact removal above) and two regressors of interest: event boundaries, and within-event control timepoints. The two analytical methods used were ROI-based, and voxelwise (with FDR correction). In the ROI analyses, average percent signal change from baseline (nuisance regressors + non-modeled timepoints) was extracted from each ROI, for each participant. These values were correlated with age, and were broken up by group for simple group comparisons via one-way ANOVA and followup post-hoc Tukey's HSD. Voxelwise analyses were computed using AFNI's 3danova function, and event boundary timepoints were explicitly contrasted with control within-event timepoints.
Effect(s) tested	Boundary-evoked activity was operationalized as activity where the percent signal change during event boundaries differed significantly from the baseline term, and within-event activity was operationalized similarly except for timepoints in the middle of events. Both ROI-based analyses and voxelwise analyses considered boundary-evoked activity in contrast to within-event activity (i.e., activity related to event boundaries while accounting for activity present within an event).

Specify type of analysis: ☐ Whole brain ☐ ROI-based ☒ Both

Anatomical location(s)	ROIs were gathered from the Desikan FreeSurfer atlas, as well as medial temporal lobe atlases which can be obtained using the following link: https://neurovault.org/collections/3731/
Statistic type for inference (See Eklund et al. 2016)	Voxelwise analyses were used in a confirmatory analysis to supplement and verify our focus on particular ROIs.
Correction	Voxelwise analyses were FDR corrected as part of AFNI's 3danova function, and significant voxels exceeded a corrected threshold of $p < 0.05$.

Models & analysis

n/a	Involved in the study
☒	☐ Functional and/or effective connectivity
☒	☐ Graph analysis
☒	☐ Multivariate modeling or predictive analysis
